# Moral competence, moral teamwork and moral action - the European Moral Case Deliberation Outcomes (Euro-MCD) Instrument 2.0 and its revision process

**DOI:** 10.1186/s12910-020-00493-3

**Published:** 2020-07-02

**Authors:** J. C. de Snoo-Trimp, H. C. W. de Vet, G. A. M. Widdershoven, A. C. Molewijk, M. Svantesson

**Affiliations:** 1 Amsterdam UMC, Vrije Universiteit Amsterdam, Department of Medical Humanities, Amsterdam Public Health research institute, De Boelelaan, 1117 Amsterdam, the Netherlands; 2 Amsterdam UMC, Vrije Universiteit Amsterdam, Department of Epidemiology and Biostatistics, Amsterdam Public Health research institute, De Boelelaan, 1117 Amsterdam, the Netherlands; 3 Center for Medical Ethics, University of Oslo, Oslo, Norway; 4 Faculty of Medicine and Health, University Health Care Research Center, Örebro University, Örebro, Sweden

**Keywords:** Clinical ethics support, Moral case deliberation, Evaluation research, Outcomes, Instrument revision, Revision process, Mixed methods

## Abstract

**Background:**

Clinical Ethics Support (CES) services are offered to support healthcare professionals in dealing with ethically difficult situations. Evaluation of CES is important to understand if it is indeed a supportive service in order to inform and improve future implementation of CES. Yet, methods to measure outcomes of CES are scarce. In 2014, the European Moral Case Deliberation Outcomes Instrument (Euro-MCD) was developed to measure outcomes of Moral Case Deliberation (MCD). To further validate the instrument, we tested it in field studies and revised it. This paper presents the Euro-MCD 2.0 and describes the revision process.

**Methods:**

The revision process comprised an iterative dialogue among the authors as Euro-MCD-project team, including empirical findings from six Euro-MCD field-studies and input from European experts in CES and theory. Empirical findings contained perceptions and experiences of MCD outcomes among healthcare professionals who participated in MCDs in various settings in Norway, Sweden and the Netherlands. Theoretical viewpoints on CES, literature on goals of CES and MCD and ethics theory guided the interpretation of the empirical findings and final selection of MCD outcomes.

**Results:**

The Euro-MCD 2.0 Instrument includes three domains: Moral Competence, Moral Teamwork and Moral Action. Moral Competence consists of items about moral sensitivity, analytical skills and virtuous attitude. Moral Teamwork includes open dialogue and supportive relationships and Moral Action refers to moral decision-making and responsible care. During the revision process, we made decisions about adding and reformulating items as well as decreasing the number from 26 to 15 items. We also altered the sentence structure of items to assess the current status of outcomes (e.g. ‘now’) instead of an assumed improvement over time (e.g. ‘better’) and we omitted the question about perceived importance.

**Conclusions:**

The Euro-MCD 2.0 is shorter, less complex and more strongly substantiated by an integration of empirical findings, theoretical reflections and dialogues with participants and experts. Use of the Euro-MCD 2.0 will facilitate evaluation of MCD and can thereby monitor and foster implementation and quality of MCD. The Euro-MCD 2.0 will strengthen future research on evaluation of outcomes of MCD.

## Background

Clinical ethics support (CES) services aim to help healthcare professionals in dealing with ethically difficult situations. These situations can occur on a daily basis and may involve personal doubts or team disagreements on what is good care in a certain situation. CES is offered in various forms, for instance through individual ethics consultants who can be called in for ethical guidance or advice [1], ethics committees who may discuss the situation as a group of experts, give advice or develop policies [2], or moral case deliberations (MCD) where a facilitator chairs a group dialogue among healthcare professionals about an ethically difficult situation with the use of a specific method [3].

In the last decades, CES services have become a common service in many healthcare settings. In North-European countries, especially in the Netherlands, MCD is a predominant type of CES [4]. MCD, also named as ethics case reflection [5], ethics rounds [6] and ethics reflection groups [7], concerns a group dialogue among healthcare professionals on a moral question about a concrete difficult situation from their practice [3, 8, 9]. The dialogue usually takes about 45–90 min and is led by a facilitator. The facilitator does not provide any advice regarding what should be done in the particular case, as expertise and moral wisdom is considered to be present among the participants themselves [10]. Participants are encouraged by the facilitator in digging for, finding and formulating an answer to the moral question, by clarifying relevant facts and perspectives, reflecting upon one’s own and each other’s viewpoints and deliberating about possible consensus and ways of acting. During this process, participants should have equal space for having a say and the reflection should stay connected to the facts of the situation [3]. Various conversation methods and facilitation styles exist to structure the process [3, 11, 12].

Evaluation of CES is important in order to know whether CES reaches the presumed goals of supporting healthcare professionals. Evaluation research is also needed to get a better understanding of the value of CES, which may contribute to monitor and foster its implementation (i.e. providing time, people and space) [13, 14]. Furthermore, ethics support staff are increasingly asked to demonstrate the impact of CES in order to justify their position within the healthcare system [13, 15–17]. Another reason for evaluation research is to further reflect upon and improve the quality of CES itself [18]. Empirical evidence for the impact of CES in general is scarce [13, 15]. CES is both rather novel as well as complex in its nature as described by Schildmann and colleagues [13]. It involves multiple interactions between various actors at different levels (i.e. personal, professional and organizational); it requires specific expertise and can be targeted to various groups, both in and outside the hospital [13]. Since CES is used in various forms and for various purposes, it may result in a variety of possible outcomes and it might be difficult to determine how a specific form of CES leads to a specific outcome [9, 13, 19]. Hence, uncertainty exists on how to establish the link between method of CES (i.e. MCD) and actual outcomes in daily practice [13, 18, 19].

As a response to the need for valid methods for evaluation research, the European Moral Case Deliberation Outcomes (Euro-MCD) instrument was developed by some members of our project team (BM, GW and MS) to assess outcomes of MCD [19]. The Euro-MCD Instrument presents a wide range of possible outcomes and asks participants to rate both importance and experience of these outcomes [19]. The presented outcomes in the Euro-MCD Instrument were based on an explorative literature review, a Delphi-expert panel and content validity testing [19].

Recently, we conducted several field studies (see Table 1) using the Euro-MCD Instrument to assess whether healthcare professionals perceived the presented outcomes as important, to examine their experiences of outcomes and to examine the clustering of items of the instrument [20–24] (De Snoo-Trimp J.C., Molewijk A.C., Svantesson M., Widdershoven G.A.M., De Vet H.C.W: Field-testing the Euro-MCD Instrument: Important outcomes according to participants before and after Moral Case Deliberation. Submitted). Based on this process of field-testing, time is ripe to present a revision of the Euro-MCD instrument.

**Table 1:** Overview of studies for revision process Euro-MCD Instrument (2014)

Study		Focus	Methods	Key findings
I	De Snoo-Trimp et al. 2017 [20]	Perceived importance of outcomes before participation in MCD	Mixed methods – Euro-MCD Instrument closed responses from Dutch healthcare professionals and interviews with Dutch healthcare professionals	Outcomes referring to Enhanced Collaboration, Improved Moral Reflexivity and Concrete Results were perceived as most important. Quality of care was noted as extra possible outcome in interviews
II	Svantesson et al. 2019 [21]	Perceived importance of outcomes before participation in MCD	Mixed methods – Euro-MCD Instrument closed and open responses of healthcare professionals in the Netherlands, Sweden and Norway	Outcomes referring to Enhanced Collaboration and Concrete Results were perceived as most important Better interaction with patient and family members was mentioned as extra possible outcome in open answers
III	De Snoo-Trimp et al. 2020	Analyzing structure and stability of items on perceived importance before and after MCD participation	Quantitative – Euro-MCD Instrument closed responses of healthcare professionals in Netherlands, Sweden and Norway and factor structure	Outcomes referring to Enhanced Collaboration and Moral Reflexivity were perceived as most important both before and after participation. Healthcare professionals found similar outcomes the most important after participating in MCD series but rated outcomes less important than prior to participation. Factor structure showed 3 domains of outcomes.
IV	De Snoo-Trimp et al. 2019 [22]	Experience of outcomes after MCD participation, both during sessions as well as in daily practice	Quantitative – Euro-MCD Instrument closed responses of healthcare professionals in Netherlands, Sweden and Norway and factor structure	Outcomes referring to Enhanced Collaboration, Moral Attitude and Moral Reflexivity were mostly experienced. Factor structure showed 4 domains of outcomes.
V	De Snoo-Trimp et al. 2018 [23]	Defining and categorizing MCD outcomes in focus group sessions with experienced MCD participants	Qualitative: focus group sessions with experienced MCD participants in the Netherlands, using method of Concept Mapping	In total, 85 possible MCD outcomes were categorized into 8 domains, of which 4 referred to individual development, 2 referred to the group level, 1 concerned the organizational level and 1 was about concrete actions.
VI	Silén & Svantesson 2019 [24]	Experienced impact what MCD meant for daily practice according to managers	Qualitative: interviews with managers from workplaces where MCD was practiced in Euro-MCD project in Sweden	The theme of enhanced ethical climate emerged as main outcome in experiences of managers.

The twofold aim of this paper is 1) to present the revised Euro-MCD 2.0 instrument, and 2) to describe the revision process.

## Methods

**Fig. 1:**
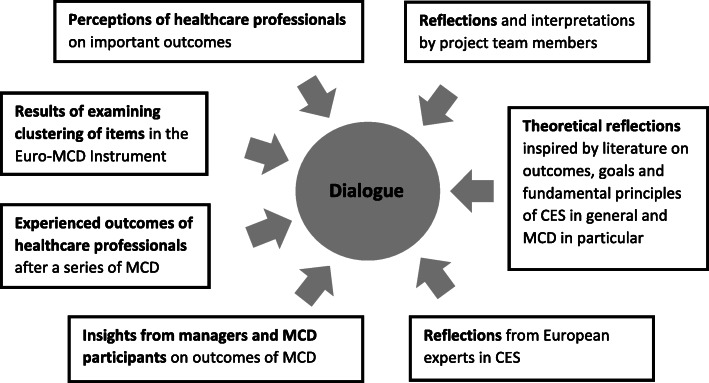
Revision process of the Euro-MCD instrument from 2014. Overview of the integration of sources. Manuscript Moral Competence, Moral Teamwork and Moral Action – The European Moral Case Deliberation Outcomes (Euro-MCD) Instrument 2.0 and its revision process

The core of the revision process of the Euro-MCD Instrument was a continuous dialogue in which we combined empirical findings with theoretical reflections, as visualized in Fig. 1. Empirical findings concerned mixed-methods field studies on the prioritized and experienced outcomes of MCD participants and the factor structure of the Euro-MCD Instrument. Theoretical reflections were based on relevant literature on outcomes, goals, ethics theory and theoretical viewpoints on CES in general and MCD in particular. In a further step, results were discussed with European experts in CES in an expert meeting. Their views were integrated in the revision process.

### The original Euro-MCD instrument from 2014

The Euro-MCD Instrument (2014-version) consisted of open and closed questions. First, two open questions were posed to respondents asking to describe important and experienced outcomes in their own words. Closed questions concerned a list of 26 possible outcomes of MCD. These 26 outcomes were classified in six domains: 1) Enhanced Emotional Support; 2) Enhanced Collaboration; 3) Improved Moral Reflexivity; 4) Improved Moral Attitude; 5) Impact on the Organizational Level and 6) Concrete Results. For each outcome, the respondent was asked to rate the degree of *experience* on a four point Likert scale, considering a) the MCD sessions and b) daily practice. The respondent was further asked to rate the *importance* of each outcome on a four point Likert scale. The option ‘Cannot take a stand’ was also offered. Lastly, the respondent was invited to prioritize the 5 most important outcomes from the list of 26 outcomes. The Euro-MCD Instrument included free space after each question for comments regarding the formulation. More details and formulation of the outcomes can be found in the development paper [19].

### Sources of data for the revision: six Euro-MCD field studies

The Euro-MCD Instrument was tested in four field studies (De Snoo-Trimp J.C., Molewijk A.C., Svantesson M., Widdershoven G.A.M., De Vet H.C.W: Field-testing the Euro-MCD Instrument: Important outcomes according to participants before and after Moral Case Deliberation. Submitted) [20–22] and reflection on MCD outcomes in general was done in two additional studies [23, 24] as shown in Table 1.

With regards to the field studies, studies I-III involve answers of respondents to the question ‘*How important is this outcome according to you?*’, collected before (studies I and II) and after (study III) participation in a series of MCD, and supplemented with qualitative data (open answers and interviews). The question ‘*To what degree did you experience this outcome?*’ was assessed in study IV, regarding experienced outcomes both during the MCD sessions and in daily practice. With regards to the additional studies, study V concerned a focus group study among Dutch MCD participants with considerable MCD experience in various healthcare settings; who were (being) trained as MCD facilitator. They brainstormed on possible outcomes of MCD and categorized these outcomes via the method of Concept Mapping [25]. In addition, items from the Euro-MCD Instrument were shown to these focus group members and – if considered relevant by them – added to the list and included in the categorization. Lastly, study VI concerned Swedish managers’ experiences of impact of MCD in daily practice, interviewed in healthcare settings where MCD had been organized.

In three studies (II-IV), healthcare professionals from various healthcare settings in Norway, Sweden and the Netherlands were invited to complete the Euro-MCD Instrument. Studies I and V were conducted among Dutch healthcare professionals only, study VI included exclusively Swedish managers. Recruitment for studies I-IV was done via healthcare institutions that planned to implement MCD on a structural basis, with possibilities to distribute the Euro-MCD Instrument among participants before and after a series of 4 to 8 MCD sessions. Recruitment for study V was done via facilitators from the national training for MCD facilitators and a Dutch network for trained facilitators. The interviewed managers from study VI were recruited via Swedish healthcare institutions that were enrolled in the field studies. The Euro-MCD Instrument was distributed on paper or by e-mail in Sweden and the Netherlands and as online questionnaire in Norway. In all field studies, participation was voluntary and based on informed consent. More details regarding recruitment procedures and respondents’ characteristics can be found elsewhere [20–24].

### Data analysis and integration

The various sources in the revision process – empirical findings, dialogues and theoretical reflections – were considered as equally important and in need of each other to revise the Instrument (see Fig. 1). The perspective of users of MCD was needed to learn what they found important to be able to manage ethically difficult situations in daily clinical practice. By collecting and analyzing their answers, we gained insight in how respondents interpreted and valued the presented outcomes of the Euro-MCD instrument. The items rated as less important, experienced in a low degree or with no associations with other items in the factor analyses, were reconsidered as they might not be sufficiently relevant or clear. As such, the empirical findings served as a guidance to delete, reformulate or combine items. Moreover, factor analyses showed if and how items could be categorized.

While empirical findings show what outcomes are experienced and perceived as important by MCD participants, this does not necessarily mean that these outcomes *should* be outcomes of MCD. Therefore, we needed to interpret and reflect on the empirical findings in the light of theories on goals and fundamental elements of MCD (and CES in general). But also vice versa: if we, based on a theory underlying MCD, would consider a certain goal to be fundamentally important for MCD, this goal should be recognizable and represented in the items of the instrument and preferably also in the empirical findings. If, for instance, respondents might consider a ‘theoretically important outcome’ as *un*important, there would be a need to understand their views or extensive justifications before including this outcome in the revision. For this, thorough and in-depth dialogues were essential.

Hence, a dialogue, including reflection on empirical findings and theoretical aspects of MCD, took iteratively place during the entire revision process. This was done through several rounds in which the members of the project team first individually and independently provided interpretations and reflections on findings and wrote proposals for revision. Subsequently, we discussed these to understand each other’s arguments and to achieve consensus (face-to-face and via digital communication). The goal was to build clear, relevant and meaningful domains: domain names should make clear for all users and readers what the items are about and the items should be valid in constructing that particular domain. Additionally, the domain should be meaningful in the sense that the name should preferably indicate the moral dimension at stake (e.g. ‘*good* care’ or ‘*moral* attitude’). We further considered for each original Euro-MCD item separately whether it was sufficiently relevant and clear and whether it needed reformulation.

### Final phase of revision: development of Euro-MCD 2.0

After extensive re-categorizations and reformulations by the project team, a first draft of domains and items of the new Euro-MCD 2.0 was presented to European experts in CES in an expert panel meeting. In this meeting, experts from a variety of CES practices participated, with expertise in both CES and CES evaluation research. Their characteristics are shown in the Appendix (part I, Table 4). In the audiotaped meeting, the experts were invited to give critical and constructive comments regarding categorization, item formulation, rationale and purposes of the revised instrument or anything else they found remarkable in this draft version. Their feedback was taken into account when further finalizing the revision and developing new drafts of the instrument. Lastly, a final draft was reviewed by and discussed with English native speakers and CES experts in think aloud interviews to check the interpretation and clarity of domains and items. Their characteristics are also presented in the Appendix (part I, Table 5).

## Results

The result of the revision process is the revised Euro-MCD Instrument: the Euro-MCD 2.0. This will be presented and explained in part I, including comparisons with the Euro-MCD Instrument from 2014. In part II, we elaborate on the revision process and our arguments for revision as developed throughout the iterative revision process.

### Part I: presenting and explaining the revised Euro-MCD instrument: Euro-MCD 2.0

The Euro-MCD 2.0 consists of 15 items covered by three domains: 1) Moral Competence; 2) Moral Teamwork and 3) Moral Action, as presented in Table 2. This table also shows the link with previous Euro-MCD items. In the Appendix (part II), the complete Euro-MCD 2.0 including instructions and answer options is presented.

**Table 2:** The Euro-MCD 2.0 - Revised Instrument

(Sub)Category	Item #	Link to former Euro-MCD item (if any)
**Moral Competence**		
Moral sensitivity	1. I recognize a situation as being ethically difficult	Increases my awareness of the complexity of ethically difficult situations (no. 12)
	2. I am aware of others’ perspectives in ethically difficult situations	I see the ethically difficult situations from different perspectives (no. 14)
Analytical skills	3. I can identify the different values at stake in ethically difficult situations	Develops my ability to identify the core ethical question in the difficult situations (no. 13)
	4. I can formulate arguments in favor of and against different courses of actions in ethically difficult situations	Find more courses of actions to manage the ethically difficult situations (no. 24)
Virtuous attitude	5. I listen with an open mind to others when discussing an ethically difficult situation	I listen more seriously to others’ opinions (no. 18)
	6. I speak up in ethically difficult situations	Strengthens my self-confidence when managing ethically difficult situations (no. 2) & Gives me more courage to express my ethical standpoint (no. 19)
**Moral Teamwork**		
Open dialogue	7. We openly express our viewpoints in ethically difficult situations	More open communication among co-workers (no. 10)
	8. We all have opportunities to express our viewpoint on ethically difficult situations	Greater opportunity for everyone to have their say (no. 6)
	9. We respect different viewpoints when discussing ethically difficult situations	Enhanced mutual respect amongst co-workers (no. 8)
Supportive relationships	10. We feel secure to share emotions in ethically difficult situations	Enhances possibility to share difficult emotions and thoughts with co-workers (no. 1) & I feel more secure to express doubts or uncertainty (no. 5)
	11. We support each other when dealing with ethically difficult situations	
**Moral Action**		
Moral decision-making	12. We make decisions on how to act in ethically difficult situations	Find more courses of actions to manage the ethically difficult situations (no. 24) & Enables me and my co-workers to decide on concrete actions in order to manage the ethically difficult situations (no. 26)
	13. We base our decisions on moral considerations in ethically difficult situations	
Responsible care	14. We are responsive to the values and needs of patients and their families when interacting with them in ethically difficult situations	
	15. We are able to explain and justify our care towards patients and their families	

#### Moral Competence

The first domain of Moral Competence includes ‘moral awareness’, ‘analytical skills’ and a ‘virtuous attitude’ when experiencing and dealing with an ethically difficult situation. In the field studies, outcomes referring to moral competences were valued and experienced by participants and associated with each other. Due to MCD, participants might develop awareness to recognize a situation as being ethically difficult (item #1) and become aware of others’ perspectives (items #2). Furthermore, participants might grow in analytical skills to identify values and formulate arguments when encountering an ethically difficult situation (items #3 and #4). Besides, a virtuous attitude can become more apparent by openness when listening to others (item #5) and courage to speak up in ethically difficult situations (item #6).

During our deliberations on this domain, literature on moral competence helped us to further reflect upon the name of this domain and refine the formulation of its items. Moral competence is a rather broad concept in literature, most often used in business ethics and theories on moral development. The three subdomains of awareness, skills and attitude have been described in several studies close to ours as possible outcomes of ethics education or ethics support. First, the three elements of awareness, skills and attitude are reflected in the focus on perception and reflection by Kälvemark Sporrong et al. [26], who argue that ethical competence ‘entails the ability to integrate perception, reflection and action, and to understand oneself as being responsible for one’s own actions’. Furthermore, Eriksson et al. [27] argued that ethical competence should include ‘being’ (i.e. virtues), ‘doing’ (i.e. acting according to ethical guidelines and rules): and ‘knowing’ (i.e. reflecting on relevant virtues and guidelines). More recently, in their development of ethics education aimed to foster moral competence, Van Baarle and colleagues [28] operationalized moral competence as follows: ‘moral competence entails the ability to be aware of one’s personal moral values and the values of others, the ability to recognize the moral dimension of situations, the ability to judge adequately a moral dilemma, to communicate this judgment, the willingness and ability to act in accordance with this judgment in a morally responsible manner, and the willingness and ability to be accountable to yourself and to others’. In this definition, the focus on awareness can clearly be recognized, and the repeated use of the words ‘willingness and ability’ reflect similar attitudes and skills as we propose in this domain.

In this domain of Moral Competence, items of the original Euro-MCD domains of Moral Reflexivity (no. 11, 12, 14 and 15), Moral Attitude (no. 16–20) and (to some extent) Emotional Support (no. 2 and 4) were reformulated and integrated. This was due to the factor analyses (studies III, IV) not supporting a distinction between them, hence, we merged them. The field studies (I-IV), both regarding perceived importance as well as experience, did nevertheless show the value of the items from these domains.

#### Moral Teamwork

The second domain is Moral Teamwork and involves two subdomains: ‘open dialogue’ and ‘supportive relationships’ among healthcare professionals. As MCD inherently is a group exercise in interaction, the MCD meetings might have an impact on how the involved healthcare professionals as a group talk and work together when facing an ethically difficult situation, also beyond the MCD sessions in their daily practice. The field studies clearly showed that outcomes about teamwork were highly valued and experienced by MCD participants. ‘Moral teamwork’ was chosen as a name for this domain. This name was considered to cover the content as closely and clearly as possible: it is not only about communication but rather about their joint way of working together, their teamwork, related to ethically difficult situations. And it is not about the practical content of this teamwork but about its *moral* aspects. The items in the subdomain of ‘an open dialogue’ involve whether team members talk openly and honestly with each other (item #7), discuss ethical issues on an equal level (item #8) and in a respectful way (item #9). The subdomain of ‘supportive relationships’ is about whether the team members feel secure amongst each other to share emotions (item #10) and motivated to support each other when dealing with ethically difficult situations (item #11).

In order to define this domain and (re) formulate its items, we used aspects from existing literature on teamwork. Literature on teamwork is extensive and terms like ‘team effectiveness’ or ‘interprofessional teamwork’ have been studied across various research areas (e.g. business, sociology, medicine) [29–31]. For instance, Schmutz et al. [31] recently examined the link between effective teams and clinical performance because they saw that ‘researchers and practitioners often lack a common conceptual foundation for investigating teams and teamwork in healthcare’. In their meta-analytical review, they defined teams as ‘identifiable social work units consisting of two or more people with several unique characteristics’. Next, they operationalized teamwork as follows: ‘teamwork is a process that describes interactions among team members who combine collective resources to resolve task demands (e.g. giving clear orders)’ [31]. They further made a distinction between ‘teamwork’ and ‘taskwork’. The latter concerns ‘*what* a team is doing whereas *teamwork* is *how* the members of a team are doing something with each other’. This distinction is helpful for us since our domain Moral Teamwork is about *how* the team members work together in ethical matters, not primarily about *what* they – in the end – do to manage the ethical situation. Furthermore, this definition is focused on the *interaction* between team members, which resembles our focus on dialogue in this domain. The focus on dialogue also appears in the definition by Babiker et al. [30] of team effectiveness: ‘an effective team is a one where the team members, including the patients, communicate with each other, as well as merging their observations, expertise and decision-making responsibilities to optimize patients’ care’. They described several characteristics of an effective team, including some with a clear link to our domain, like ‘honesty’ and ‘effective communication’ referring to open and equal interaction possibilities for team members. In addition, a literature review by Mickan & Rodger [29] revealed eighteen characteristics for ‘effective teamwork’ in healthcare, categorized into an organizational domain, a domain for the contributions of individual team members and a domain for ‘team processes’. In this latter domain, the notions of ‘communication’ ‘cohesion’, and ‘social relationships’ are relevant. These notions are reflected in our subdomains dialogue and relationships.

One major topic of discussion in the project team was whether we should call this domain ‘ethical climate’, which also focuses on dialogue and relationships [24, 32–35]. Ethical climate is mainly characterized as ‘shared perceptions’ of values and supportive relationships among healthcare professionals and the presence of possibilities to reflect, decide and act in an ethical way [32–35]. It is comparable to what MCD envisions in facilitating dialogue, mutual understanding and common grounds when dealing with ethical challenges. The project team therefore considered that MCD outcomes in the domain of Moral Teamwork show similarities with aspects of ethical climate. At the same time, ethical climate has been described to cover more than only team collaboration and is used as a rather broad concept involving both possibilities for ethical reflection (e.g. ethics consultants or MCD) as well as relationships, beliefs and behavior of individuals. This is for instance described by Silén and Svantesson [24] in their recent study on manager’s experiences with clinical ethics support, where they extensively elaborated on the concept of ethical climate. They argued that ethical climate might involve both group dynamics as well as ‘morally grounded actions and morally strengthened individuals’. In the end, we came to consensus on using ‘moral teamwork’ as a more pragmatic term, meaningful with regards to the content and at the same abstraction level as the other domain names.

The domain Moral Teamwork includes some adapted items from the former domains of Enhanced Collaboration (no. 6,8 and 10) and Emotional Support (no. 1 and 5). Since this domain is about how participants work *together*, all items are formulated as ‘We … ’. A new item (#11) is added: ‘We support each other when dealing with an ethically difficult situation’ as this mutual support was considered to be an essential element of moral teamwork and suggested by respondents in open answers of both our field studies (I-III) and our focus group study (study V).

#### Moral Action

Lastly, the domain of Moral Action involves the subdomains ‘moral decision-making’ and ‘responsible care’. The project team considered it important to include items referring to concrete decisions and actual caring practice, as was also suggested in the closed and open responses of respondents in the field studies. The deliberation in MCD might not only change the participants in their individual moral competences (the first domain) and their teamwork (the second domain), but also, and maybe even *through* the first and second domain, the actual situation itself.

Firstly, in the subdomain ‘moral decision-making’, we want to assess whether MCD participants report to make a decision on how to deal with the situation at all (item #12) and if they base these decisions on moral considerations (item #13). Making a decision on moral grounds refers to how participants perceived the deliberation: did they consider the moral aspects of the situations, and not only the medical facts or psychosocial worries? In line with the theoretical background of MCD, the deliberation ideally results in a plan of action. According to hermeneutic pragmatic philosophy and dialogical ethics, one may start to experience and understand things in a new way and come to new or adapted plans of action [10, 36]. In one of our field studies, managers of workplaces where MCD took place told that ‘ethics was more marked in written documents, such as the operational plan, in notes regarding breakpoint dialogues and care goals as well as in reasons for changing decisions’ [24]. As such, MCD seems to impact the actual daily practice and in particular how concrete decisions are made or changed.

Secondly, we built the subdomain ‘responsible care’ to indicate the relationship with patients (and their families) and to explicitly show our operationalization of ‘good’ care: depended on the context and clarified by the responsible healthcare professional. We considered that a core element of providing good care concerns a responsiveness to the values and needs of patients and their family when interacting with them (item #14). Experiencing and valuing good interactions with patients and family can be seen as a crucial element of good care, as most general ethics approaches plea for patient-centered approaches in healthcare [37]. In particular, the care ethics approach emphasizes the interdependency and equal relationships between care-givers and care-receivers [38, 39]. A care ethics approach fits well to the daily practice of healthcare – the setting where MCD takes place. Here, healthcare professionals may have complex interactions with various stakeholders, confronting them with fundamental questions challenging their own presuppositions. In addition, the patient being the most important stakeholder is often a vulnerable person, hence, the healthcare professional should establish a responsible relationship with him or her [40]. Next, we previously described that a definition on good care would not fit in the Euro-MCD Instrument, as good care is exactly what MCD participants deliberate on in the MCD session (as is the case in CES in general). Yet, the result of this deliberation should (at least) be that responsible healthcare professionals are able to explain their view on good care to patients and their families. Therefore, assessing whether good care has been reached should be focused on the process instead of the content, and on the perceptions of participants. Therefore, we could ask MCD participants whether *they think* they are able to explain and justify their care towards patients and their families, which we assess in our last new item (#15).

Items from the former Euro-MCD domain of Concrete Results (no. 24 and 26) are merged in in the subdomain of moral decision-making: ‘We make decisions on how to act in ethically difficult situations’ (item #12). In this subdomain, a new item (#13) is added: ‘We base our decisions on moral considerations in ethically difficult situations’. The items in the subdomain ‘responsible care’ are also new: ‘We are responsive to the values and needs of patients and their family when interacting with them in ethically difficult situations’ (#14) and ‘We are able to explain and justify our care towards patients and their families’ (#15).

### Part II: the revision process in detail

We will now describe how our decisions for revision were based on the empirical findings and developed throughout our revision process. First, a brief summary of the empirical findings is given, followed by a description of how these findings indicate points for revision and reflection.

#### Summary of the six field studies

In short, the following conclusions regarding the Euro-MCD Instrument (2014-version) could be drawn based on the empirical field studies:

- The majority of respondents rated all MCD outcomes as quite or very important, both before and after MCD participation, without a considerable difference between these moments (Studies I-III, Table 1).
- Outcomes referring to the domain ‘Enhanced Collaboration’ were particularly valued (Studies I-III) and experienced by the majority of respondents (Study IV)
- Outcomes regarding the domain ‘Concrete Results’ were perceived as quite or very important before MCD participation (studies I-II)
- Outcomes regarding the domain ‘Moral Attitude’ were experienced in a quite or very high degree during the sessions and in daily practice (Study IV)
- Outcomes referring to quality of care and the interaction with patients and their family members were suggested as new outcomes by respondents who were about to participate in MCD (Studies I-II)
- Factor analyses of the outcomes did not confirm the six originally proposed domains but revealed three or four domains of outcomes, indicating a possible distinction between virtues, skills, sharing feelings and actions (Study III-IV)
- Twelve outcomes of the 26 (no. 1,3,5,9,13,15,17,19,22–25 in Table 3) should be reconsidered regarding importance or clarity of formulation as these had low associations with other items in the factor analyses (Studies III-IV)
- Experienced MCD participants listed 85 possible outcomes of MCD into eight categories of which four categories referred to personal development (as professional and individual, focused on the other, knowledge and skills), two concerned the team (with regards to its development and connection), one referred to organization and policy and one referred to concrete actions (Study V)
- Outcomes reported by managers were categorized as an enhanced ethical climate, including a closer-knit team, morally strengthened professionals, morally grounded actions and ethics leaving its marks on everyday work (Study VI)

**Table 3:** Euro-MCD domains and items (2014) – Arguments for adaptation, reformulation or deletion

Domain and Item	Consideration project team	Decision
**Enhanced Emotional Support**		
1. Enhances possibility to share difficult emotions and thoughts with co-workers	Needs reconsideration, was important for respondents but might have been misinterpreted by respondents as it does not correlate with other items from the domain Emotional Support.	Rewritten as item #10 in revised domain ‘Moral Teamwork’: *We feel secure to share emotions in ethically difficult situations*
2. Strengthens my self-confidence when managing ethically difficult situations	Good item but seems to belong to Moral Attitude rather than to Emotional Support	Included in item #6 in revised domain ‘Moral Competence’: *I speak up in ethically difficult situations*
3. Enables me to better manage the stress caused by ethically difficult situations	Needs adaptation or deletion, too vague, might have been misinterpreted by respondents and managing stress might not be a necessary outcome of MCD at all	Deleted
4. Increased awareness of my own emotions regarding ethically difficult situations	Good item but seems to belong to Moral Attitude rather than to Emotional Support	Not included because of item reduction, as other items in revised domain ‘Moral Competence’ were determined as being closely related concept
5. I feel more secure to express doubts or uncertainty regarding ethically difficult situations	Needs reconsideration as it does not seem to be important according to respondents and does not seem to correlate with other items from Emotional Support and it might be too similar to items 2 and 5.	Rewritten as a group-related outcome, item #10 in revised domain ‘Moral Teamwork’: *We feel secure to share emotions in ethically difficult situations*
**Enhanced Collaboration**		
6. Greater opportunity for everyone to have their say	Good and important item	Included as item #8 in revised domain ‘Moral Teamwork’: *We all have opportunities to express our viewpoint on ethically difficult situations*
7. Better mutual understanding of each other’s reasoning and acting	Good and important item, but might need reconsideration as it correlates with both individual items (5 and 19) and group items (6,8 and 10) indicating various possible interpretations.	Deleted because of item reduction as it was considered to be covered by other items
8. Enhanced mutual respect amongst co-workers	Good item but might need reconsideration as it also seems to correlate with items from Moral Attitude.	Rewritten as item #9 in revised domain ‘Moral Teamwork’: *We respect different viewpoints when discussing ethically difficult situations*
9. I and my co-workers manage disagreements more constructively	Needs reconsideration or deletion as it does not seem to be important or experienced according to respondents indicating that it might not be an outcome of MCD at all.	Deletion
10. More open communication among co-workers	Good and important item	Included as item #7 in revised domain ‘Moral Teamwork’: *We openly express our viewpoints in ethically difficult situations*
**Improved Moral Reflexivity**		
11. Develops my skills to analyse ethically difficult situations	Needs reconsideration – might be too general and already covered by other items	Deleted because of item reduction as it was considered to be covered by other items
12. Increases my awareness of the complexity of ethically difficult situations	Good and important item	Rewritten as item #1 in revised domain ‘Moral Competence’: *I recognize a situation as being ethically difficult*
13. Develops my ability to identify the core ethical question in the difficult situations	Needs reconsideration or deletion as it does not seem to be important according to respondents and it might be too similar to other items from Moral Reflexivity.	Changed and rewritten as item #3 in revised domain ‘Moral Competence’: *I can identify the different values at stake in ethically difficult situations*
14. I see the ethically difficult situations from different perspectives	Good and important item	Included as item #2 in revised domain ‘Moral Competence’: *I am aware of others’ perspectives in ethically difficult situations*
15. Enhances my understanding of ethical theories (ethical principles, values and norms)	Needs adaptation or deletion, as it might not be an outcome of MCD at all	Deleted as it was not considered to be relevant/intended outcome of MCD
**Improved Moral Attitude**		
16. I become more aware of my preconceived notions	Good item but might be too general considering the correlations with many other items and possible social desirability in its formulation	Deleted because of item reduction, not considered to be a clear outcome of MCD.
17. I gain more clarity about my own responsibility in the ethically difficult situations	Needs reconsideration or deletion as it might have been misinterpreted as shown by the lack of correlations with other items in the perceived importance-data.	Deleted as it was not considered to be a clear outcome of MCD
18. I listen more seriously to others’ opinions	Good item but might need reconsideration as it seems to become important for respondents only after participation in MCD.	Rewritten as item #7 in revised domain ‘Moral Competence’: *I listen with an open mind to others when discussing an ethically difficult situation*
19. Gives me more courage to express my ethical standpoint	Needs reconsideration as it does not seem to be important according to respondents and it might be too similar to items 2 and 5.	Deleted because item about self-confidence was considered as same outcome, item #6 in revised domain ‘Moral Competence’: *I speak up in ethically difficult situations*
20. I understand better what it means to be a good professional	Good item but might be too general considering the correlations with many other items and possible social desirability in its formulation	Deleted because of item reduction, too vague and general formulation
**Impact on Organizational Level**		
21. I and my co-workers become more aware of recurring ethically difficult situations	Needs reconsideration since the item seems to be about moral reflexivity than the organizational level regarding the correlations with items from the Moral Reflexivity domain.	Deleted because of item reduction and too vague to apply to experience *before* MCD participation.
22. Contributes to the development of practice/policy in the workplace	Needs adaptation or deletion, might have been misinterpreted by respondents or developing policies might not be an outcome of MCD at all	Deleted, not necessarily an outcome of MCD
23. I and my co-workers examine more critically the existing practice/policies in the workplace/organization	Needs reconsideration or deletion as it does not seem to be important or experienced according to respondents indicating that it might not be an outcome of MCD at all.	Deleted because of item reduction and too vague to apply to experience *before* MCD participation.
**Concrete Results**		
24. Find more courses of actions to manage the ethically difficult situations	Needs reconsideration, seems to be important for respondents but might have been misinterpreted by respondents as it does not seem to correlate with other items from the domain Concrete Results.	Included as item #4 in revised domain of ‘Moral Competence’: *I can formulate arguments in favor of and against different courses of action in ethically difficult situations*
25. Consensus is gained amongst co-workers in how to manage the ethically difficult situations	Needs adaptation or deletion, too vague as it does not seem to belong to domain of concrete results	Deleted due to item reduction and being too vague
26. Enables me and my co-workers to decide on concrete actions in order to manage the ethically difficult situations	Good and important item	Included as item #12 in revised domain of ‘Moral Action’: ‘*We make decisions on how to act in ethically difficult situations’*

A detailed overview of the results and considerations *per Euro-MCD item* is presented in Table 3.

Based on the field studies, the following decisions for revising the Euro-MCD Instrument were made: 1) reformulating items and changing all items into assessing the current status of MCD related outcomes (e.g. ‘now’) instead of change over time (e.g. ‘better’); 2) changing the original domains; 3) adding items about quality of care and interacting with patients and family; 4) omitting the question about perceived importance; and 5) deleting items not sufficiently relevant to or associated with MCD.

##### Reformulating items to assess current instead of changed practice

Firstly, the formulation of items turned out to be problematic in the field studies. All outcomes were formulated in a comparative manner including words like ‘more’ or ‘better’, for instance: ‘More open communication among co-workers’ or ‘I understand better what it means to be a good professional’. This could have made it rather straightforward for respondents to agree on their importance and difficult to disagree with them. Moreover, potential bias might have occurred here as respondents might be directed towards desirable answer options regarding their practice. It could also have made it hard for respondents to discriminate between items regarding both importance as well as experience. Therefore, the decision was made to reformulate outcomes more neutrally and about the current practice instead of a transition or indication of an improvement, like ‘We openly express our viewpoints in ethically difficult situations’ (#7). As a result of this reformulation, we changed the answer options as well, from a *degree* of importance or experience towards an *agreement* on the item, on a four point Likert scale from ‘strongly agree’ to ‘strongly disagree’.

##### Changing the original domains

A second point for revision that emerged from the empirical findings concerned the categorization of outcomes. As described before, the original Euro-MCD Instrument consisted of 6 domains. These domains were not confirmed in the factor structures of the data, as factor analyses revealed 3 and 4 domains for the perceived-importance and experience question respectively. In particular, the domains Impact on the Organizational Level and Concrete Results needed reconsideration since their items were not associated with each other and did thus not convincingly form distinct domains. Therefore, we left these six domains and made a new categorization in the revised instrument, in which elements of these former domains can still be recognized. Initially, consensus was reached on a general division of items on the individual, group and case level. This division was indicated by the factor analyses. The first level referred to individual development and changes due to participation in MCD, including awareness, skills and attitude. The second level comprised the impact on dialogue and relationships among healthcare professionals as a group or team and the third level was linked to actual care practices and decisions made about the concrete quality of care. The next step was to go from abstract levels to definite domains including items. We have described this in the previous part.

##### Adding items about quality of care and interaction with patients and family

Furthermore, a point for revision was the consideration of new items, like quality of care (as suggested in study I) and better interaction with patient and family (as suggested in study II). With regards to quality of care: we considered that contributing to quality of care is the ultimate and overarching goal of clinical ethics support. In the end, MCD should support healthcare professionals to pursue high quality of patient care. At the same time, it has been described to be complicated to give concrete and universal definitions of quality of care in general, and more specific as outcome of CES since CES inherently concerns a reflection upon how we define quality of care [3, 41]. Subsequently, it is difficult or maybe impossible to directly define the impact of ethics support on quality of care [6, 13, 15]. Therefore, a predefined outcome regarding what quality of care should look like does not fit here.

This does however not mean that it is not at all possible to link MCD to quality of care, as it is at least possible to assess how healthcare professionals *themselves* think about the process to arrive at good decisions, or how they think about preconditions to deliver good care. As MCD is mostly intended to be a service supporting healthcare professionals in defining good care, it is important that outcome measures stay close to how professionals define good care. In the end, outcomes referring to quality of care, like all outcomes in the Euro-MCD Instrument, should only be included if healthcare professionals are able to recognize and experience them. Support for this could be found in the focus group study (study V), in which items referring to the *procedure* to arrive at good care were suggested, such as ‘Clarify what good care entails’ and ‘Better quality of work’. We further reflected on these suggestions when defining items in the new domain of ‘Moral Action’, see part I.

##### Omitting the question about perceived importance

Fourthly, the question on perceived importance of the presented MCD outcomes needed reconsideration. Since respondents perceived all outcomes as quite or very important, without a meaningful change over time, there was no clear emphasis on or discrimination between certain outcomes. The reason for these high rates is not clear. Perhaps MCD might have been very welcome as opportunity to sit and talk, − in particular – for Scandinavian nurse assistants, which might partly explain why outcomes were rated so high in the Scandinavian countries (study II). In the end, we concluded that the question on perceived importance would not have any value in the revised version because the field study respondents confirmed their assumed relevance and did not discriminate between items to allow for tailoring or weighing outcomes. It is however important to note that the question has been of great value in the revision process as it showed the perceptions of end-users regarding the relevance and importance of items.

##### Deleting items not sufficiently relevant to or associated with MCD

Some items of the Euro-MCD Instrument were omitted (see Table 3), due to a lack of correlations with other items or low experience-rates in the empirical data, implying to be insufficiently relevant or associated with MCD. The item ‘Enables me to better manage stress caused by ethical difficult situations’(no. 3), was believed to have a vague formulation. Also, we concluded that some items with low scores or low correlations (no. 9, 13,22 and 25) did not appear to be clear outcomes of MCD. Firstly, we decided to delete the item ‘I and my co-workers manage disagreements more constructively’ (no. 9). Although we considered it as a relevant outcome that participants might learn to deal with disagreements during and after MCD, it might have been too ambitious to learn this after a few MCD sessions. It might also have been too difficult to answer as it requires thinking about both disagreement itself as well as with how disagreement is dealt. Next, we considered learning about ethical theory (no. 13) not as a characteristic for the process of MCD as MCD is not a theoretical course but a reflective dialogue focusing on participants’ perspectives. The item about developing practice and policy (no. 22) was not considered as basically relevant for healthcare professionals and might have been a too ambitious goal of participating in some MCD sessions. Lastly, gaining consensus (no. 25) did not seem to be interpreted as a ‘Concrete Results’-outcome by respondents. We concluded that the term ‘consensus’ is confusing: does it mean that everyone agrees on the decision? Does it relate to shared decision-making, in the sense that all relevant parties should be involved in the decision-making process? In the end, MCD is not per se about decision-making or a joint agreement, and important parties for decision-making like patients or family might be absent. Therefore, we decided to delete this item. Nevertheless, aspects from these outcomes on how healthcare professionals jointly discuss about and decide on ethically difficult situations are resembled in the revised instrument (see part I).

#### Finalizing the instrument

In the last phase of the revision process, the draft version was discussed with four native English speakers in think aloud interviews, resulting in clarifications and adjustments on detailed item level. (See the Appendix I, Table 5 for their characteristics.) One of the suggestions was to divide the experience in the MCD sessions from the experience in daily practice, by making two separate questionnaires for each setting, with the same items. We accepted this suggestion as it was considered to enhance the readability and feasibility for future users of the Euro-MCD Instrument. As a consequence, respondents now have to rate their experience for only one setting (MCD sessions or daily practice). We decided that the Euro-MCD 2.0 can be completed at three moments: 1) at baseline, so before MCD participation, to assess experience of the listed outcomes in current daily practice; 2) directly after (a series of) MCD, to assess experience of outcomes *during* these MCD(s) and 3) at a later moment after (a series of) MCD, to assess experience of outcomes in daily practice. In the introduction of the questionnaire, respondents are instructed which setting they should consider when rating the items. As such, the context in which the respondent completes the instrument determines the particular question for the 15 items: if we want to know the outcomes with regard to the sessions, we ask about their experience when thinking about the sessions. If we want to know the outcomes with regard to daily practice (either before or after MCD participation), we ask respondents to complete the questionnaire with their daily practice in mind. In finalizing the instrument, we checked whether items were applicable for all these moments.

## Discussion

This paper presents the Euro-MCD 2.0, as well as the arguments developed in the revision process. We already described our reflections on the Euro-MCD 2.0 and the field studies in the Results section, as this was part of the revision process. We will now further reflect on the revision process *itself*, by describing our methodological considerations, including strengths and weaknesses. Furthermore, we here provide an outlook to future research on and application of the Euro-MCD 2.0.

### Methodological considerations about the revision process

Our dialogical approach to revise the Euro-MCD Instrument is in line with one of the approaches to evaluate CES as described by Schildmann and colleagues [42]. In their approach of ‘reconstructing quality norms’, they describe that criteria for evaluation of CES only become clear through deliberation among CES participants within specific contexts: ‘outcomes are defined by the stakeholders in the practice [i.e. the end users] in close cooperation with CES [Service] experts and researchers’. Therefore, during our revision process, we explicitly included the perspectives of MCD participants in the field studies and invited experts from various European settings where CES is applied. An ongoing dialogue among various researchers and MCD participants required open, transparent, extensive and regular meetings to keep on track regarding the presumed goal of revising the instrument. A challenge of the revision process was the lack of a clear protocol on how to start and the steps to follow. As there was no established method or example for developing an evaluation tool in this field of research, neither for integrating empirical findings with theoretical reflections, the current process of revision was a pioneering exercise.

In the revision process, the dialogue was not limited to the research members only since the empirical findings can be seen as a dialogical ‘partner’ as well and we received input from experienced MCD participants in one of our field studies [23] and feedback from European experts in the field of CES. The latter feedback was also important to create broader support from experts with various expertise and from different European countries for the Euro-MCD 2.0 as an actual *European* instrument. In the revision process, we constantly searched for a way to construct possible outcomes of MCD that refer to ‘good’ healthcare professionals, working together in a ‘good’ way to contribute to ‘good’ care or ‘good’ decisions. In this process, we operationalized this ‘good’ in the new domains and subdomains, as for instance shown in the name ‘responsible care’. At the same time, we took care to leave room for the deliberative and reflective nature of MCD regarding what this ‘good’ should be in concrete situations.

One of the strengths of our approach was the multidisciplinary and multinational variety in all parts and phases. We used various quantitative and qualitative methods and involved respondents from a wide range of healthcare settings and professional backgrounds in different countries. Furthermore, diverse interpretations of the data occurred, dependent on the MCD contexts we knew and were used to (e.g. Swedish ethics reflection groups in community care or Dutch moral case deliberations in emergency settings) and the research methodologies we were familiar with, ranging from instrument development to interview studies and philosophical analysis. Due to this variety, project team members were challenged to explain and provide arguments for their own viewpoint and to listen to others’ suggestions. This was an intensive process involving many and lengthy structured and well-documented meetings in the project team about proposals for revision which were individually and independently prepared by the project team members. The combination of various data sources was also a strength since the sources confirmed and justified decisions regarding the revision. For instance, the final structure into three domains is similar to both the suggested division by the factor analyses (on perceived importance) as well as the categorization by MCD participants in the concept mapping study.

Some weaknesses should be mentioned. In the revision process, only a limited number of countries were explicitly involved. As a consequence, empirical findings for the revision were based on only Swedish, Norwegian and Dutch data. Furthermore, the project team for this revision consisted only of one non-Dutch researcher. However, ethics support experts from Sweden and other countries (UK, Germany, Switzerland) were involved in the final phase. We assume that the instrument is feasible for MCD practices in other countries and settings as well. Yet, this applicability of the instrument is not confirmed yet. Another weakness was that given a broad definition of MCD, we were not able to show which components of MCD contribute to the outcomes, as was recently indicated as field of inquiry by Schildmann and colleagues [13]. In our field studies, we did not have information about how the MCD sessions were performed, hence, we were not able to relate any specific component of MCD to the outcomes. At the same time, we (and others) did study the content of MCD in the settings where the Euro-MCD Instrument was distributed [9, 40, 41, 43, 44]. These studies show that MCD is a space for moral reasoning, reflections on context, relieving emotions, sharing uncertainties and concerns about a situation, and that the role of facilitator is deemed as crucial. Yet, these studies did not examine the link between these components and the outcomes of MCD. We therefore recommend further research into the link between content and outcomes of MCD to improve quality of both MCD itself as its impact.

### Recommendations for future use of the Euro-MCD 2.0

The Euro-MCD 2.0 can be used in healthcare settings where MCD is implemented as a service to support healthcare professionals in handling ethical challenges. We want to stress here that we do not claim that all outcomes will be or should be affected by MCD. The instrument includes possible outcomes of MCD and can provide a detailed overview of how participants experience *possible* MCD outcomes. As such, organizations can be informed on outcomes in order to foster and adjust structural implementation of MCD. Also, facilitators might get insight into possible points for improvement of their role and the way they use and steer the MCD sessions when learning about how outcomes are experienced and possibly developed over time. The Euro-MCD 2.0 further allows for comparison of experienced outcomes between and within diverse professional groups or healthcare teams in order to tailor the service of MCD to these specific groups and settings. Moreover, the focus in the formulation of the items is now on the current practice instead of any self-reported changes. As such, the Euro-MCD 2.0 can monitor possible developments in outcomes by comparing the status quo with the status after some MCDs: did MCD participants grow in their competences, teamwork and care? Lastly, the Euro-MCD 2.0 was and is initially developed for MCD, but might be applicable for other types of CES evaluation research as well. This applies in particular to CES services where a dialogue takes place about a moral question that has risen from a specific situation and where relevant perspectives, values and norms are considered. The Euro-MCD 2.0 could also be used in other settings than healthcare, yet pilot-testing and eventual adapted formulations of items will then be needed.

Next, it might be interesting to compare results of the Euro-MCD with results from other relevant measurement instruments like quality of care measures, moral distress scales and ethical climate scales in order to assess whether (for instance) positive ratings for experiencing outcomes of MCD are associated with higher scores on one of the other scales. As such, the Euro-MCD Instrument can contribute to the need for ‘further rigorous research to evaluate the effectiveness of ethical case interventions’ [14]. We therefore recommend comparing various measurement tools, scales and instruments in future evaluation research in the field of ethics support.

Furthermore, since participation of patients and their family members in MCD is a growing area of interest [45–47], we recommend participatory research studies to also explore patients’ views on outcomes of MCD. Lastly, apart from a few ‘think aloud’ interviews, the current structure and content of the Euro-MCD 2.0 have not been tested yet. So along the use of the instrument in future studies and in clinical practice, it is important to collect data for future validation of the Euro-MCD 2.0.

## Conclusions

The Euro-MCD 2.0 is shorter and less complex than the original Euro-MCD Instrument: the number of items is reduced from 26 to 15 items and the number of domains from six to three. It is now more strongly substantiated by an integration of empirical data from several field studies, theoretical reflections and ongoing dialogues with MCD participants and European experts in CES and evaluation. The instrument determines whether healthcare professionals have experienced possible MCD related outcomes regarding their moral competences, moral teamwork and moral action. Through this, the Euro-MCD 2.0 can assess if and how MCD supports healthcare professionals in dealing with ethically difficult situations, both during the MCD sessions as well as in daily practice. The instrument can now be used in various healthcare settings to improve MCD in clinical practice. As a tool for evaluation, the Euro-MCD 2.0 may help to monitor, foster and when needed adjust the implementation and quality of MCD or other CES services, which aim to support healthcare professionals in dealing with ethically difficult situations and striving towards better care.

## Data Availability

Not applicable. All data used for this study has been published in our previous field studies [20–24].

## Appendix A

**Table 4:** Expert meeting with Ethics support experts May 2019 – Characteristics (*N* = 8)

**Countries (N)** • Germany (1) • The Netherlands (2) • Sweden (3) • Switzerland (2)	
**Professional background (N)** • Philosophy (4) • Nursing (3) • Medicine (1)	
**Researcher position (N)** • Junior researcher (2) • Senior researcher (3) • Associate professor (3)	
**Familiar with Euro-MCD Instrument (2014) (N)** • Yes, used it (4) • No (4)	

**Table 5:** Participants Think Aloud interviews (*N* = 4)

**Countries (N)** • The Netherlands (1) • United Kingdom (2) • United States (1)	
**Professional background (N)** • Ethics (2) • Nursing (1) • Social Science (1)	
**Familiar with Euro-MCD Instrument (from 2014) (N)** • No (4)	

## Appendix B

**Table 6:** The Euro-MCD Instrument 2.0^a^

Instruction: Please rate the extent to which you agree on the following statements, when thinking about your daily practice/the MCD session(s) that you participated in.					
	Strongly agree	Slightly agree	Slightly disagree	Strongly disagree	I don’t know
**1st Category: Moral Competence** *Moral Sensitivity*					
1. I recognize a situation as being ethically difficult					
2. I am aware of others’ perspectives in ethically difficult situations					
*Analytical Skills*					
3. I can identify the different values at stake in ethically difficult situations					
4. I can formulate arguments in favor of and against different courses of action in ethically difficult situations					
*Virtuous attitude*					
5. I listen with an open mind to others when discussing an ethically difficult situation					
6. I speak up in ethically difficult situations					
**2nd Category: Moral Teamwork** *We = the people with whom you have participated in the MCD session(s)/the people with whom you work in your daily practice.* *Open Dialogue*					
7. We openly express our viewpoints in ethically difficult situations					
8. We all have opportunities to express our viewpoint on ethically difficult situations					
9. We respect different viewpoints when discussing ethically difficult situations					
*Supportive Relationships*					
10. We feel secure to share emotions in ethically difficult situations					
11. We support each other when dealing with ethically difficult situations					
**3rd Category: Moral Action** *Moral decision-making*					
12. We make decisions on how to act in ethically difficult situations					
13. We base our decisions on moral considerations in ethically difficult situations					
*Responsible care*					
14. We are responsive to the values and needs of patients and their families in ethically difficult situations					
15. We are able to explain and justify our care towards patients and their families					

^a^Please contact us for a user-friendly version, updates and/or translated versions (Dutch, Swedish) via j.desnoo@a amsterdamumc.nl

## Abbreviations

CES: Clinical Ethics Support
Euro-MCD: European Moral Case Deliberation Outcomes
MCD: Moral Case Deliberation
